# A 1000-Year Carbon Isotope Rainfall Proxy Record from South African Baobab Trees (*Adansonia digitata* L.)

**DOI:** 10.1371/journal.pone.0124202

**Published:** 2015-05-13

**Authors:** Stephan Woodborne, Grant Hall, Iain Robertson, Adrian Patrut, Mathieu Rouault, Neil J. Loader, Michele Hofmeyr

**Affiliations:** 1 iThemba LABS, Private Bag 11, Wits 2050, South Africa; 2 Mammal Research Institute, University of Pretoria, Private Bag X20, Hatfield 0028, South Africa; 3 Department of Geography, Swansea University, Swansea SA2 8PP, United Kingdom; 4 Faculty of Chemistry, Babes-Bolyai University, Arany Janos 11, 400028 Cluj-Napoca, Romania; 5 Nansen-Tutu Center for Marine Environment, University of Cape Town, Cape Town, South Africa; 6 Dept of Oceanography, Mare Institute, University of Cape Town, Cape Town, South Africa; 7 SANParks Scientific Services, Pvt Bag X402, Skukuza, 1350, South Africa; University of California San Diego, UNITED STATES

## Abstract

A proxy rainfall record for northeastern South Africa based on carbon isotope analysis of four baobab (*Adansonia digitata* L.) trees shows centennial and decadal scale variability over the last 1,000 years. The record is in good agreement with a 200-year tree ring record from Zimbabwe, and it indicates the existence of a rainfall dipole between the summer and winter rainfall areas of South Africa. The wettest period was c. AD 1075 in the Medieval Warm Period, and the driest periods were c. AD 1635, c. AD 1695 and c. AD1805 during the Little Ice Age. Decadal-scale variability suggests that the rainfall forcing mechanisms are a complex interaction between proximal and distal factors. Periods of higher rainfall are significantly associated with lower sea-surface temperatures in the Agulhas Current core region and a negative Dipole Moment Index in the Indian Ocean. The correlation between rainfall and the El Niño/Southern Oscillation Index is non-static. Wetter conditions are associated with predominantly El Niño conditions over most of the record, but since about AD 1970 this relationship inverted and wet conditions are currently associated with la Nina conditions. The effect of both proximal and distal oceanic influences are insufficient to explain the rainfall regime shift between the Medieval Warm Period and the Little Ice Age, and the evidence suggests that this was the result of a northward shift of the subtropical westerlies rather than a southward shift of the Intertropical Convergence Zone.

## Introduction

**Fig 1:**
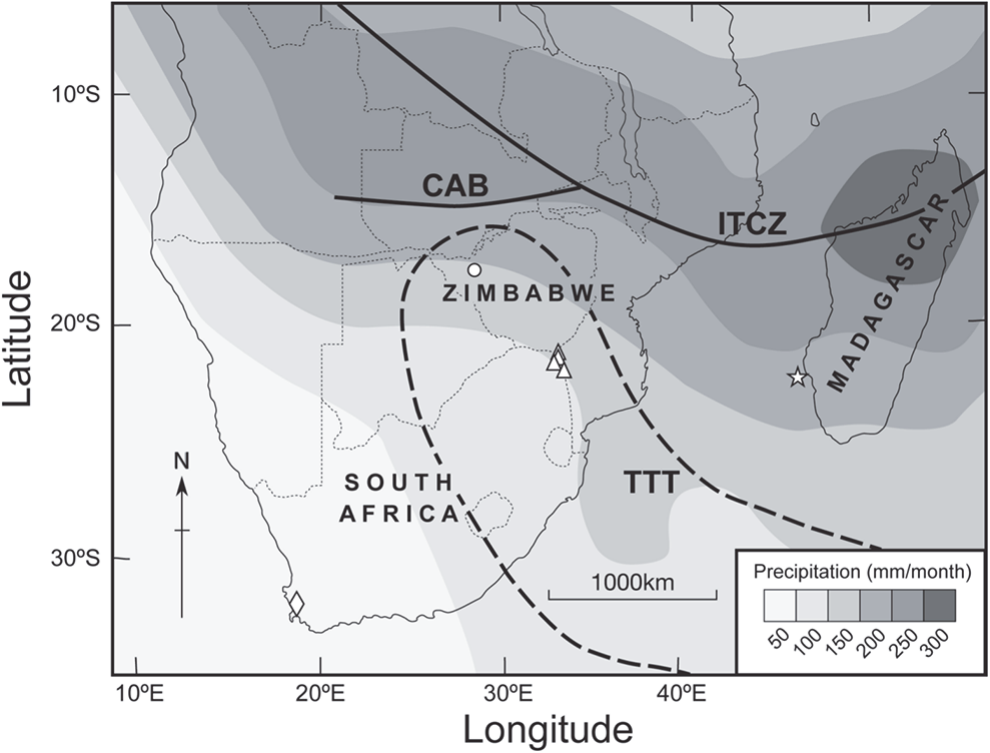
Sample sites and austral summer rainfall density over southern Africa. The highest rainfall density is associated with the southernmost position of the intertropical convergence zone (ITCZ) (solid line) after reference [2]. The approximate position of the TTT system is within the dotted line after reference [4]. Sampling sites and reference data sites include: the baobab trees (triangles), mukwa trees from western Zimbabwe (circle), the cedar chronology (diamond), and the sea-surface temperature for the Mozambique Channel is derived from the coral isotope record from Ifaty (star).

Much of the Southern African rainfall is of convective origin forced by large-scale dynamics during the Austral summer [1]. The summer rainfall region experiences dramatic inter-annual changes leading to severe droughts or floods that affect agricultural productivity, particularly subsistence farming, as well as water reserves. The region lies to the south of the intertropical convergence zone (ITCZ) [2] and modal rainfall is derived from temperate-tropical troughs (TTT) [3]. East/west displacement of the TTT system plays an important role in modulating rainfall amount [1, 4] (Fig 1). Studies of the limited instrumental records from the region, and modeled data, suggest that the seasonal position of this climate system is influenced by remote ocean atmosphere interactions such as the El Niño/Southern Oscillation (ENSO). The relationship between ENSO and rainfall is non-linear and it has become stronger since the 1970s but prior to this correlations are poor [5, 6]. Most severe droughts occur during the mature phase of El Niño [6, 7]. The Indian Ocean Dipole Moment Index (IOD) [8] or (DMI) [9] also has an influence on rainfall. Droughts are associated with a positive phase of the IOD [10]. In addition the Southwestern Indian Ocean (Mozambique Channel and Agulhas Current system) is the main source of moisture for precipitation in southern Africa [11] and local sea surface temperatures (SST) also affects rainfall [6]. In contrast to East Africa where rainfall is strongly linked to SST in the Indian Ocean [12, 13], the correlation between rainfall in southern Africa and Southwestern Indian Ocean SST is reported to be weaker than it is with ENSO or the IOD [6].

The ENSO, IOD and SST variability are oceanic drivers that may influence rainfall in southern Africa on the inter-annual to decadal time scales through synoptic circulation responses. Over millennial or centennial time scales the climate systems may respond to external forcing such as the Maunder solar minimum (Little Ice Age). Modeling of the effect of northern Hemisphere cooling suggests that the ITCZ moved southwards during the Little Ice Age [14, 15]. Evidence of dry conditions in Lake Edward [16] and wetter conditions in Lake Challa and Lake Naivasha [12] have been interpreted as evidence for this shift over East Africa. Southward shifts of the ITCZ during the Little Ice Age have also been reported from other regions of the world [17, 18, 19]. This supports millennial scale palaeo-data suggesting that southern African rainfall may be forced by northern hemisphere influence on the position of the ITCZ, and the role of Indian Ocean SST is only an enabling factor [20]. However the effect of oceanic cooling in the Agulhas Current region leads to northward shifts in the subtropical westerlies [21, 22]. Research on rainfall changes in the winter rainfall region of southern Africa, which received rain from frontal systems in the westerlies, suggests that the westerlies moved northward during the Little Ice Age [17, 23, 24, 25]. As the TTT system forms between the ITCZ in the north and the westerlies in the south, the millennial scale rainfall variability in TTT derived rainfall is likely to respond to these latitudinal changes in the climate systems.

While the relationships between oceanic conditions and rainfall in southern Africa appear to be coherent, forecasting skill for their influences on precipitation is poor. Instrumental records of climatic and SST variability that may elucidate the ocean/climate dynamics under long-term forcing are lacking [26]. Palaeo-precipitation proxies, in particular, lack the required precision and temporal or spatial resolution to provide useful comparisons with palaeo-SST records. A high-resolution rainfall reconstruction is required to elucidate the dynamic evolution of the underlying climate forcing in the region.

Palaeo-precipitation reconstruction using traditional dendroclimatological approaches in southern Africa is limited because few tree species have been shown to form annual rings or rings that accurately record climatic parameters in their widths [27, 28]. Chronologies have been developed using mukwa trees (*Pterocarpus angolensis*) in Namibia [29], Botswana [28] and Zimbabwe [30] but the longest of these records is 201 years. Chronologies based on wild seringa trees (*Burkea africana*) [29] and msasa trees (*Brachystegia spiciformis*) [31] in Namibia and Zimbabwe are of shorter duration. The only chronology from southern Africa that extends far enough back to record the influence of the Maunder solar minimum is based on cedar trees (*Widringtonia cedarbergensis*) from the southwestern Cape of South Africa [32], which is in the winter rainfall area.

An alternative approach to palaeoclimate reconstruction that may be useful when tree ring widths are not a tenable proxy lies in the physiological control that trees exert on carbon assimilation. In particular the physiological response to water stress affects the carbon isotope ratios (δ^13^C) in wood [33, 34]. The uptake of carbon is dominated by a biochemical reaction involving Rubisco fixation that has a constant fractionation effect on the carbon isotope composition of the wood relative to atmospheric CO_2_. A second effect is stomatal conductance, which is controlled by the relative availability of edaphic water (rainfall) and external leaf water vapor deficit (humidity). This is what allows wood δ^13^C to potentially proxy rainfall as the photosynthetic binding of atmospheric CO_2_ discriminates against ^13^CO_2_ in favor of ^12^CO_2_ [33]. During periods of high water availability the leaf stomata open and allow rapid exchange of CO_2_ with the atmosphere, and the discrimination against ^13^CO_2_ takes place in an open system. During periods of low water availability the stomata close to reduce water loss, and the CO_2_ available for photosynthesis is limited to that inside the leaf. As the internal leaf CO_2_ concentration decreases the concentration of ^13^CO_2_ increases and the photosynthetic products reflect the increased ^13^C content. This constrains which species and specimens may be used in this approach. Only specimens that are dependent on precipitation as a water source are suitable. For trees that grew away from watercourses and are also shallow rooted, the dominant driver of carbon isotope variability is soil moisture, which is directly linked to rainfall amount. The framework suggests that, in regions of low rainfall, as is the case in southern Africa, lower δ^13^C values are associated with wetter conditions, and higher δ^13^C values are associated with dryer conditions during the growth of the wood [34].

The theoretical basis for a carbon isotope chronology approach has been empirically verified at a local scale and also at a regional scale [35]. In South Africa carbon isotope chronologies of coastal red milkwood trees (*Mimusops caffra*) [36], real yellowwood trees (*Podocarpus latifolius*) [37] and matumi trees (*Breonadia salicina*) [38] have been correlated with past climates. Carbon isotope ratios of growth rings in African baobab trees (*Adansonia digitata*) are also shown to be significantly correlated with summer precipitation (r = 0.72, p<0.01)[39] but caution must be applied in the interpretation of this study as it is based on an extralimital specimen grown in a cultivated garden setting in which rainfall is not the only source of edaphic water. The great age to which baobab trees persist [40, 41] offers the potential to reconstruct a long palaeo-precipitation record for southern Africa. We integrate the carbon isotope records from 4 baobab trees from the Limpopo River Valley (Fig 1) that grew over the last 1000 years in order to produce a regional proxy rainfall record.

## Materials and Methods

### Baobab sampling

Baobab trees were sampled in conservation areas of South Africa with permission from SANParks. Two trees that died naturally in the Pafuri area yielded samples that were large enough to separate individual ring structures. These are named the Pafuri-4 baobab (22° 22.925'S, 31° 13.145'E) and the Pafuri Outpost baobab (22°26.647' S, 031°04.745' E) and they are important because they are the only trees for which the innermost (juvenile) rings were recovered. A further 3 cores were taken from 2 living baobab trees, named the Lebombo Eco Trail baobab (23°15.765' S, 031°33.309' E) and the Pafuri-1 baobab (22°24.352' S, 031°16.676' E). Cores were removed using a Haglöf CO600 increment borer (60 cm long, 0.43 cm inner diameter) in which distortion and compression prevented the separation of individual rings. The great size of these trees excludes any possibility that the borer was able to sample the center of the trees. Three of the trees are in close proximity to one another, with the Lebombo Eco Trail tree located c. 100km to the south (Fig 1). All of the specimens were situated outside of drainage features. The trees are from the summer rainfall region of southern Africa, in an area of high drought risk [42].

### Baobab chronology

AMS radiocarbon analyses of the baobab cores were performed on 1cm long samples that were forfeited from the isotopic time series analysis (Table 1). Radiocarbon dates were calibrated using the Southern Hemisphere dataset [43]. The midpoint of each 1cm section was assigned the most likely intercept values to generate the initial chronology. The dating of the Lebombo Eco Trail baobab has been central in developing an understanding of the growth dynamics and architecture of baobab trees [44]. The evidence suggests that baobabs occasionally cease to lay down growth rings for extended periods of time. All of the samples presented in this study, with the exception of one, show evidence for stop or stop/start growth (S1 Fig). Although stop/start growth is a common feature, the radiocarbon analyses on individual cores demonstrates that growth periods are radially linear.

**Table 1:** Radiocarbon dates.

Tree	Core/ Ring#	Length (cm)*	Sample duration (years)	Date (Uncalibrated years BP)	Laboratory #	Assigned year (AD)
Lebombo Eco Trail	2	7.5	10.3	850±22	OS-84375	1223
	2	15.5	10.3	971±26	OS-84388	1140
	2	16.5	10.3	992±31	OS-87713	1130
	2	29.5	10.3	1043±24	OS-85870	996
	7	22.5	12	222±30	OS-85869	1736
	7	45.5	12	435±26	OS-87450	1451
Pafuri-4	68	10	1	138.5±0.4pmc	OS-89543	1977
	570	85	1	337±27	OS-89515	1564
	734	160	1	624±24	OS-89541	1400
Pafuri Gila Pan	3	7.5	2.7	118.8±0.4pmc	OS-84397	1987
	3	18.5	2.7	102.1±0.3pmc	OS-84396	1956
	3	25.5	10.2	697±24	OS-87447	1370
	3	37	10.2	871±23	OS-84448	1186
Pafuri Outpost	27	Not noted	1	368±20	Pta-9813	1511
	40	Not noted	1	412±20	Pta-9814	1498
	293	Not noted	1	806±20	Pta-5555	1360
	490	Not noted	1	1000±20	Pta-9797	1154

The assigned age for each radiocarbon date falls within the 1-sigma calibrated error.

*Length measurements along cores originate at the surface of the trees

The core sections were subdivided into equal size aliquots for the isotopic analysis, and each aliquot was attributed an age based on the linear interpolation of the initial radiocarbon determinations. For the samples with identifiable growth rings, ^14^C analysis on individual rings established that temporal resolution is consistent with Robertson et al. [39] who have shown that baobab rings form annually in this region. The age model was therefore based on this understanding (S1 Fig). The presumption that rings form annually is a conclusion that is made specifically for these specimens, as the physiology of baobab growth has not yet been elucidated. Under different conditions, for example where rainfall seasonality is bimodal, the growth structures may differ. One of the dates from the Lebombo-4 baobab is inconsistent with annual ring formation, but this derives from a buttress formation in which ring structures were thicker and not easily identified. Here a linear interpolation was used for the buttressed section of the stem.

Isotopic excursions were represented in all of the trees, and based on the initial chronology it was apparent that slight adjustments in the age allocations would reconcile patently similar isotopic oscillations in different trees. On this basis the most likely calibration intercept was rejected and the age attributed to each AMS analysis was adjusted within the 1-sigma uncertainty range. This approach allowed similar isotopic excursions to occur concurrently between records.

### Isotopic analysis

The residual cores (after removal of dating aliquots) were subdivided into equal sized sub-aliquots with resolution dictated by the sample size requirements of isotopic analysis pre-treatment and measurement. The average length of each aliquot was 1.9mm (range 1.5mm – 2.6mm). Taking the age model into account the average aliquots represents 1.9 years (range 0.8 years to 3.3 years). Aliquots were pre-treated to α-cellulose [45] and the carbon isotopes measured at the CSIR, Pretoria, South Africa, on a DeltaV isotope mass spectrometer coupled with a Flash EA 1112 series elemental analyser by a Conflo IV. An in-house wood standard (*Shorea superba*) [36, 37] and blank were run at the start and end of each analysis, and after every 12 unknown samples. Precision of standards was <0.2‰. The isotopic time series from each baobab was adjusted for temporal changes of δ^13^C in the atmosphere [46], with updates available online http://cdiac.ornl.gov/ftp/db1014/isotope.cgo.

### Statistics

The isotopic time series includes errors derived from averaging between trees and temporal errors. In addition there is a great deal of variability that derives from the climate system and in particular the spatial heterogeneity of precipitation from convective cloud systems. It is only during large-scale events, such tropical cyclone *Eline* that made landfall in the Limpopo River Valley in AD 2000 [47], that all trees in the region will experience sub-seasonal (event scale) common forcing. This is the basis for inter-tree correlations in the age model development and it is verified by the fact that all the baobab growth corresponding to AD 2000 has very low δ^13^C values (indicative of wet conditions). However the precipitation from convective storms is typically spatially heterogeneous and for any storm the rainfall experienced at one location may be substantially different from another. When averaged over a rainfall season the heterogeneity in rainfall is smoothed, but not eliminated. The high frequency variability within each of the baobab δ^13^C chronologies represents the “weather” experienced by each tree and this is of less significance than the modal changes that represent the “climate” or the common forcing on all the trees.

In order to suppress the influence of localized weather anomalies and the temporal errors in the baobab record, the 21-year biweight δ^13^C mean [48] was calculated. The biweight mean is a useful time series statistical method that yields the modal value for any point in time by taking into account the record prior to, and post the data point under consideration. The duration of the prior and post periods of integration are user-defined and are selected based on the temporal resolution of the data to be analyzed, its stationarity, and the degree of proxy variability. In this case the decade before and after the year in question was considered a reasonable integration period for a climate signal without suppressing the non-stationary component in the time series. It also compensates for the temporal errors in the age models. The mean value is calculated using a weighting distribution that emphasizes the years immediately before and after the target year, and deemphasizes the contribution of points further dislocated in time. A control factor determines the extent to which the weighting reduces with values of 9 being more inclusive of the “edge” years (and the result begins to approach a running average in which high frequency variability is suppressed) and a value of 3 emphasizing the central years in the 21 year range (the resulting record retains more of the high frequency variability of the original dataset). When applied to the δ^13^C record from the baobabs we used a control factor of 9, which emphasizes the decadal trend.

The Climate Explorer website (http://climexp.knmi.nl) was used to correlate biweight δ^13^C mean time series data from the baobabs with those of ENSO and oceanic palaeotemperatures that were also subject to the biweight mean transform. This calculation is based on a Monte Carlo method for determining confidence ranges and takes the autocorrelation of the time series into account in the calculation of p-values.

## Results and Discussion

### Chronology

The age models used for the different trees and core samples are presented in S1 Fig. The use of an interpolated radiocarbon chronology is not uncommon for isotopic analysis of trees [38], but it introduces a degree of temporal error. The sampling method imposes a further element of complexity in the chronology, and a distinction is made between the trees that yielded individual ring samples, and the cores that were subdivided into aliquots for the isotope analysis. The presumption that the rings are annual helps to constrain the age argument. The ring count matches the radiocarbon age within the 1-sigma error range and the latitude for adjusting the age model is constrained by the 1-sigma range of all the radiocarbon dates simultaneously. It is estimated that the absolute error of the chronology is less than ±5 years although there is no verification that baobab trees do not produce false or missing rings. While the chronology for the two trees for which individual rings were identified is good, this does not apply to the core samples.

In the case of the core samples a linear growth model is not constrained by the radiocarbon dates simultaneously. Instead the age of each endpoint of the linear interpolation can be changed independently provided they remain within the 1-sigma range. This exaggerates the range of ages that can be assigned to any aliquot in the climate reconstruction. The inherent error is exacerbated by the extent to which a linear growth model overestimates the time frame during rapid growth periods (presumably wet years) and underestimates the time frame during slow growth (presumably dry years). The magnitude of this effect is not quantified here, and it is certain that it will be an issue when the trees grow disproportionately fast in one direction during buttress formation. In addition each carbon isotope aliquot in the core samples will potentially represent a different age stride depending on the length of the subsamples. Based on the linear growth model the duration sampled by each aliquot varies between 0.8 and 3.3 years for the subsampled cores, but this will be modulated by the variable growth rate effect.

**Fig 2:**
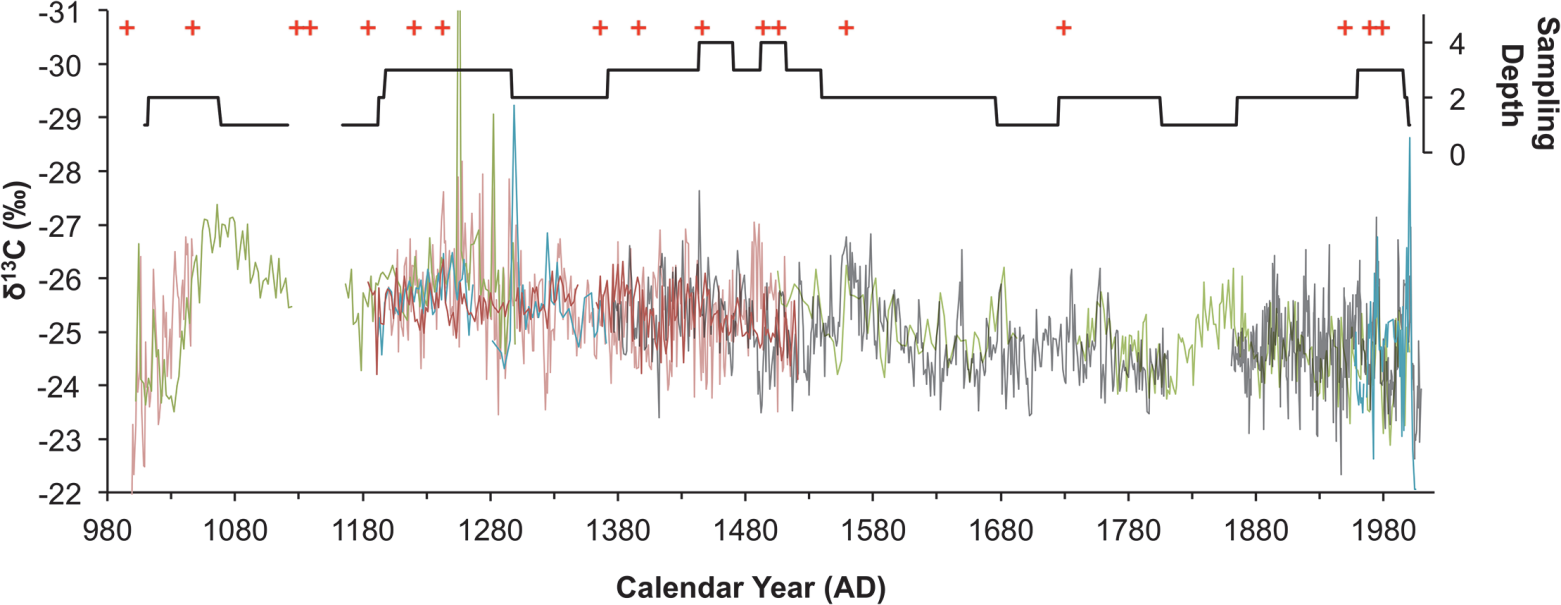
Sampling depth, dates and isotopic analysis of baobabs. The isotopic analysis of 2 baobab ring sequences and two additional baobabs yielding three core samples provides coherent variability over the last 1000 years. Each colour represents a different tree or core. The sampling depth is presented on the right axis. Red crosses indicate the age of AMS radiocarbon dates used to establish the chronology.

**Fig 3:**
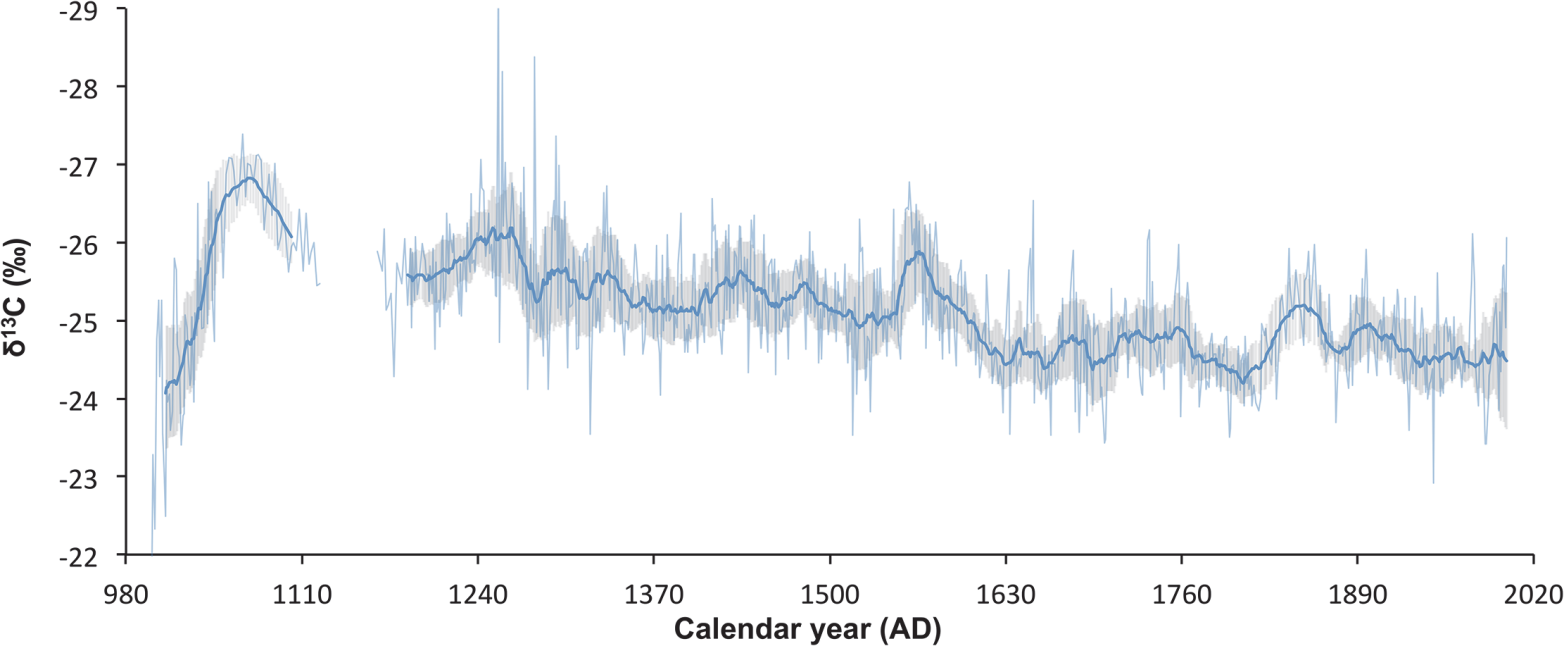
Baobab carbon isotope chronology. The δ^13^C record is averaged for 4 baobab trees (inverted y-axis portraying high (low) rainfall at the top (bottom) of the figure). The 21-year biweight mean of the isotope record (bold blue) suppresses the inter-annual rainfall variability and highlights the decadal and centennial trends.

The uncertainty in the age model differs for each of the cores in the study. When the δ^13^C values of the individual cores and ring sequences (Fig 2) were annualized to produce the isotope chronology (Fig 3) the value assigned to any particular year is the average of several samples each with a different time integral period as well as chronological errors associated with intrinsic radiocarbon errors and with the linear age interpolation. The age model is anchored on the annually resolved data from the Pafuri and Pafuri Outpost trees and has an age error of ±5 years, but for any individual δ^13^C data point from the core samples it is expected that the greatest source of error will result from differential growth rates. Since the core data points may be time averaged up to 3.3 years per point (which is assigned a single average date for the amalgamation) it is not reasonable to use this dataset to address precipitation changes at less than decadal scales.

Of the 17 dates that contribute to the chronology all remain within the 1-sigma error range of the radiocarbon analysis. A gap in the record from AD 1119 – AD 1166 represents the section of one of the cores that was used for the radiocarbon analysis.

### Isotope record

It has been shown empirically that trees that grow in different edaphic or micro-environmental conditions (slope aspect for example), but under the same climatic regime will retain the same inter-annual trend in their isotope values, but the absolute values may differ [49]. Compensation for this offset between different trees may be possible if the carbon isotope values are transformed into z-scores with a mean of zero and a standard deviation of 1 dimensionless units, but this was not possible in this study as each tree yielded a different age range in a non-stationary record. The two trees that yielded annual ring samples, the Pafuri Outpost tree and the Pafuri-4 tree, together covered the age span from AD 999 to AD 2009 with a 118-year overlap period from AD 1336—AD 1514. The period of overlap yielded a δ^13^C offset of 1.8‰. The Pafuri-4 tree is the only specimen in the sample that grew on a rocky hummock while all the others grew on sandy plains. Pine trees growing in similar circumstances show constant δ^13^C offsets [49] and, assuming that edaphic water availability underpins the offset, all the values of the Pafuri-4 tree were adjusted by 1.8‰. The combined record from the Pafuri-4 and Pafuri Outpost trees overlapped with all the other trees and in a similar fashion all their values were adjusted to a common mean for overlapping sections. In this way each tree was referenced against the Pafuri Outpost tree. Offset values for the other trees were -0.08‰ for the Lebombo Eco Trail tree and -0.04‰ for the Pafuri Gila Pan tree. There is a high degree of coherence between the isotope chronologies from the different trees (Fig 2). The record was composited by averaging the isotope values from each tree on an annualized scale (Fig 3). The 21-year biweight mean value, and biweight variance of the composited record, reflect the decadal trend in the proxy record (Fig 3) (data can be accessed at http://ncdc.noaa.gov/paleo/study/17995).

The isotope records from the Pafuri Outpost and Pafuri-4 trees that included juvenile growth rings were coherent with synchronic values from other trees and they were not excluded from the analysis.

### Rainfall proxy verification

**Fig 4:**
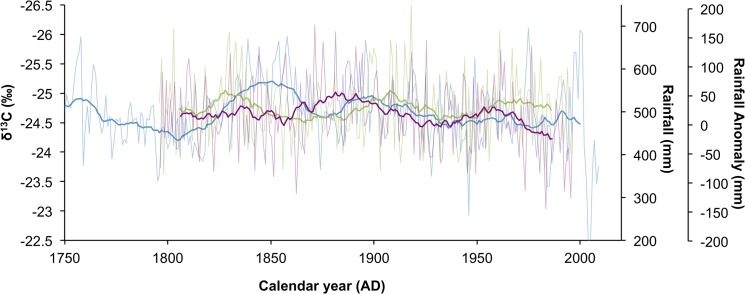
Regional rainfall proxy comparisons for southern Africa. Comparison between the δ^13^C baobab record (blue, left axis) and a rainfall record from Zimbabwe based on mukwa tree ring widths (green,1^st^ right axis) [30], and the precipitation reconstruction of Neukom et al. [5] (purple, 2^nd^ right axis). The unfiltered (pale lines) and biweight mean values (bold lines) are presented for each dataset.

In order to verify that the baobab δ^13^C record is a proxy for precipitation, it is necessary to compare it with the available rainfall records from the region. It has already been argued that it is inappropriate to compare the annualized δ^13^C record with an annualized rainfall record because of imprecision in the age model, and it is not surprising that the correlation with data from nearest rainfall station at Pafuri (22.450°S, 31.311°E), which has a near-complete record since AD 1924, is marginally significant (r = -0.246, p = 0.024, n = 84)(S2 Fig). The short duration of the instrumental record is inadequate to perform a 21-year biweight mean transform and so the baobab record is compared with the biweight mean of the rainfall reconstruction of Neukom et al. [5] and the mukwa tree ring-based rainfall reconstruction from Zimbabwe [30] (Fig 4). In both instances there is a significant correlation with the baobab record for the 20^th^ Century (r_Zim_ = -0.493, p<0.001, n = 87; r_Neu_ = -0.550, p<0.001, n = 87) but this drops to a weak but significant correlation when the 19^th^ Century is included (r_Zim_ = -0.112, p = 0.154, n = 162; r_Neu_ = -0.514, p<0.001, n = 162) (S1 Table). The theoretical expectation that δ^13^C values in trees should proxy rainfall forecasts that lower δ^13^C values should be associated with wet conditions and higher δ^13^C values should be associated with dry conditions, which is in agreement with the negative correlation obtained. The deviation between the baobab and the comparative records is largely the result of a wet period registered in the baobabs between AD 1840–1860. Even with the possible temporal errors that have been discussed it is unlikely that this wet departure could be attributed to a chronological error, and it is more likely that this is a regional rainfall variation.

### Decadal scale oceanic forcing of rainfall

**Fig 5:**
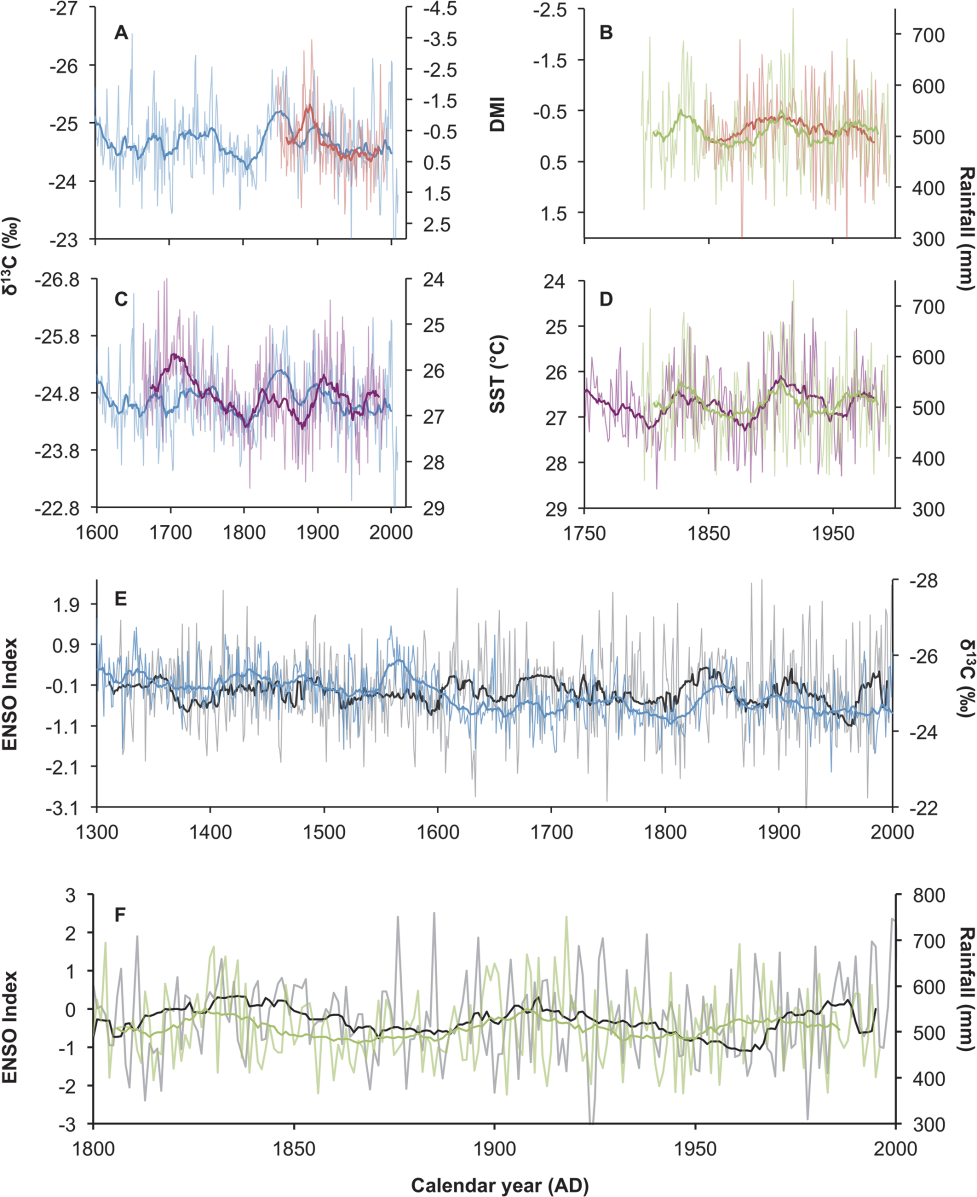
Oceanic forcing of Southern African palaeorainfall. **(A)** Periods characterised by a low Indian Ocean Dipole Moment Index (DMI) (red, inverted central axes) correlate with periods of high rainfall in the baobab record from South Africa (blue), and (B) the mukwa tree ring record from Zimbabwe. (C) Low sea-surface temperatures in the Agulhas Current core region (purple, inverted central axes) are associated with high rainfall in the baobab record and (D) in the mukwa record. (E) High rainfall in the Pafuri area is associated with El Niño conditions over most of the ENSO record [54] (grey, left axis), but specifically in the periods AD 1301-AD 1550 and AD 1710 – AD 1970 (blue, right axis). (F) ENSO also has a significant correlation with the mukwa record from Zimbabwe (green, right axis).

The baobab δ^13^C record is characterized by decadal as well as centennial variability. It indicates a shift from wetter to dryer conditions around AD 1600. Maximum rainfall occurred c. AD 1075 with minima in c. AD 1635, c. AD 1695 and c. AD1805 (Fig 3). In order to elucidate the underlying cause for the decadal variability we compare the baobab biweight mean δ^13^C and Zimbabwe mukwa records with palaeo-proxies of three oceanic mechanisms that have been linked to recent rainfall variability. These are the ENSO phenomenon which proxies SST dynamics in the Niño3,4 region in the Pacific Ocean, the DMI phenomenon in the Indian Ocean [8] as well as the SST in the Agulhas Current core area (Fig 5A–5F). There are several palaeo-reconstructions for the ENSO phenomenon: the 1100-year high-resolution reconstruction from North American droughts [50], two tree ring reconstruction of 700 years [51] and 273 years [52], and the 458 year multi-proxy record of Braganza et al. [53]. Caution must be exercised when using these ENSO proxies as there are inconsistencies in the way in which they are calculated. The indices of Stahle and Braganza are inverted relative to the instrumental Niño 3.4 record but when this is taken into account the records all provide a reassuringly coherent record of past ENSO variability (S3 Fig). The updated record of Li et al. [54] covers most of the duration of the baobab record, and we make use of this in assessing the influence of ENSO on southern African precipitation. We make use of the DMI reconstructed from coral records for the period AD 1846–1995 [9] and the SST reconstruction from Ifaty in the Mozambique Channel [55] that is argued to represent the SST in the Agulhas Current core region [56]. Correlation statistics between the biweight mean record of baobab and these parameters are summarized in S1 Table.

The dependence of rainfall on the Indian Ocean Dipole Moment (DMI) has been studied using short instrumental records from southern Africa and ensemble reconstructions [5]. The results suggest that the DMI is the dominant precipitation forcing mechanism in Zimbabwe where droughts are associated with positive states of the DMI [57, 58] while in South Africa the correlation is much weaker [5]. The coral isotope values used to reconstruct SST and derive the DMI [9] are sub-annually resolved, and so it is possible to differentiate seasonal fluctuations in the DMI. The late summer (AMJ) DMI yields the strongest correlation with the baobab record (r = 0.784, p<0.001, n = 124) (Fig 5A). The correlation between the Zimbabwe mukwa record and the DMI supports the observations of Manatsa et al. [57, 58], and the strongest correlation is also with the late summer state of the DMI (r = -0.559, p<0.001, n = 128) (Fig 5B).

While the upwelling of cold water in the eastern Indian Ocean basin exerts the strongest controls on the DMI, the counterpart SST in the western basin also plays a role. There is a strong negative relationship between the DMI and both summer (r = -0.538, p<0.001, n = 128) and winter (r = -0.638, p<0.001, n = 128) SST in the Mozambique Channel [55], which implies that seasonal cooling (warming) in the eastern Indian Ocean basin is associated with cooling (warming) in the southwestern Indian Ocean. Accordingly the influence on rainfall in southern Africa that is attributed to the DMI may be redundant with a proximal SST influence. Surface heat flux over the Agulhas Current can increase by 200 Wm^-2^ causing a 25% increase is humidity and the formation of convective cells [59]. The propagation of advected moisture over the subcontinent is dependent on wind fields and recent data indicates that there is a positive correlation between SST in the Mozambique Channel and rainfall in South Africa [60] especially during periods when the effect of ENSO is suppressed [6]. This is not supported by the baobab record. Once again the seasonal evolution of SST that is available from the sub-annual isotopic analysis of corals [55] suggests that decadal scale periods of higher rainfall are associated with cooler summer SST conditions in the Agulhas Current core area (r = 0.283, p<0.001, n = 295) (Fig 5C). Zimbabwe rainfall is strongly correlated with annualized SST in the Agulhas Current core area but the correlation is marginally stronger with the winter SST (r = -0.610, p<0.001, n = 178)(Fig 5D). Richard et al. [6] and Mulenga et al. [61] elucidate the mechanism underlying the effect. Warm SST anomalies in the southwestern Indian Ocean drive a weakening of the subtropical high belt in the southern Indian Ocean with an eastward shift of the TTT convective belts. The moisture-bearing easterly winds that normally penetrate over the subcontinent during the austral summer are no longer present and instead they effectively contribute to enhanced convective precipitation over the ocean.

The dominant influence in the recent southern African rainfall record has been ENSO [6, 7], but the relationship has been non-stationary [5, 62]. The coincidence of severe droughts with El Niño events has been strongest since the 1970s [6] but when a 200-year rainfall reconstruction was compared with an ENSO reconstruction it showed an inversion of the correlation during the 19^th^ Century [5]. Comparison between the baobab record and the updated ENSO reconstruction of Li et al. [54] shows a similar inversion: over most of the record dry conditions are associated with la Nina conditions, while wet conditions are associated with El Niño conditions (Fig 5E). In the period AD 1301 to AD 1550, before the transition to a dryer regime, the correlation between ENSO and rainfall variability is highly significant (r = -0.495, p<0.001, n = 240). Between AD 1710 and AD 1970, after the shift to a dryer regime, the correlation is also highly significant (r = -0.428, p<0.001, n = 2420). The regime transition around AD1600 and the recent past are therefore anomalous in the record. A similar pattern emerges in a comparison between the ENSO reconstruction of Li et al. [54] and the Zimbabwe rainfall reconstruction [31] (Fig 5F). Prior to AD 1950 higher rainfall is strongly correlated with predominantly El Niño conditions (r = 0.681, p<0.001, n = 126). This suggests that the current association between El Niño and drought conditions in southern Africa may be a recent phenomenon.

The methodology that has linked ENSO with rainfall requires closer scrutiny. The relationship elucidated in the baobab record is based on the 21-year biweight mean correlation, and periodically the 21-year biweight mean variability is phase-locked with the year-to-year variability of ENSO and rainfall. That is: the year-to-year rainfall variability strongly reinforces the biwieght mean with wet (dry) conditions occurring in El Niño (la Nina) years. However there are also periods in both the baobab record and the mukwa record from Zimbabwe during which the year-to-year rainfall is not phase-locked with the 21-year biweight mean trend. In these years drought (wet) conditions are associated with El Nino (la Nina) conditions in the Pacific Ocean. This might be expected as it has already been shown that the IOD and Agulhas Current SST also strongly influence rainfall. It is possible that these influences modulate ENSO. Furthermore the effect of ENSO is propagated into the Indian Ocean with lead times of up to a year [63]. As the summer rainfall region of southern Africa falls mainly in the November to March period spanning two calendar years, the annualized rainfall may not fall into the same temporal categories used in the derivation of the oceanic proxies. Correlations between ENSO and rainfall proxies from southern Africa must therefore consider lead and lag times that may exceed the temporal scale of ENSO events. This effect brings into question the approach used in many studies in which years of drought or high rainfall are compared with the corresponding ENSO state [6, 10]. The consistency of the El Niño /drought relationship that emerges from this approach is not causative because some strong El Niño (la Nina) years are not associated with drought (wet) conditions. It may emerge that the pattern observed in the baobab record will reconcile with the recent analyses if the approaches are standardized.

### Centennial scale oceanic forcing of rainfall

While the SST differentials in the Pacific Ocean and Indian Ocean that underpin the ENSO and DMI indices may contribute to the decadal variability in southern African rainfall through zonal reorganization of the equatorial Walker circulation, it is not sufficient to explain the centennial scale variability recorded in the baobab record. Modification of the Indian Ocean Walker circulation in response to east Indian Ocean SST and the Indo-Pacific Warm Pool (IPWP) has been linked to precipitation changes in East African palaeoclimate [12]. SST reconstructions for the Makassar Strait [64, 65], which is the throughflow region connecting the western Pacific Ocean with the eastern Indian Ocean in the global thermohaline circulation, show a temperature minimum at AD 1715 when Lake Challa and Lake Naivasha in East Africa show high level stands [12]. Cooling of throughflow water into the eastern Indian Ocean may suggest a strengthening of the DMI, and based on the correlation between rainfall and the DMI this would lead to the decreased rainfall in southern Africa that is evident in the baobab record. Unfortunately the coral-based DMI reconstruction does not extend back to the Medieval Warm Period to test this proposition. However the limited duration SST reconstruction for the Agulhas Current core area [57] shows that the temperature trajectory in the western Indian Ocean matches the IPWP changes [64, 65] in both timing and relative temperature changes (S4 Fig). This suggests that there was no enhancement of the DMI though time. It also suggests that the cooling that took place in the IPWP between AD 1175–1715 probably also took place in the southwestern Indian Ocean. This scenario presents another apparent contradiction because the mukwa record from Zimbabwe and the baobab record from South Africa demonstrate that lower SST in the southwestern Indian Ocean enhances rainfall over the summer rainfall area of the subcontinent. A general decline in SST from AD1175-1715 should have been associated with an enhanced precipitation trend in southern Africa, but instead the trend is one of increased aridity. A possible explanation for the rainfall trajectory is a shift in the climate bands (a regime shift). Palaeo-precipitation proxies from eastern Africa suggest that the regime shift may have been a southward shift of the ITCZ, while evidence from southern Africa suggest that it may have been a northward shift of the westerlies.

### ITCZ vs. westerly influence during the Little Ice Age

**Fig 6:**
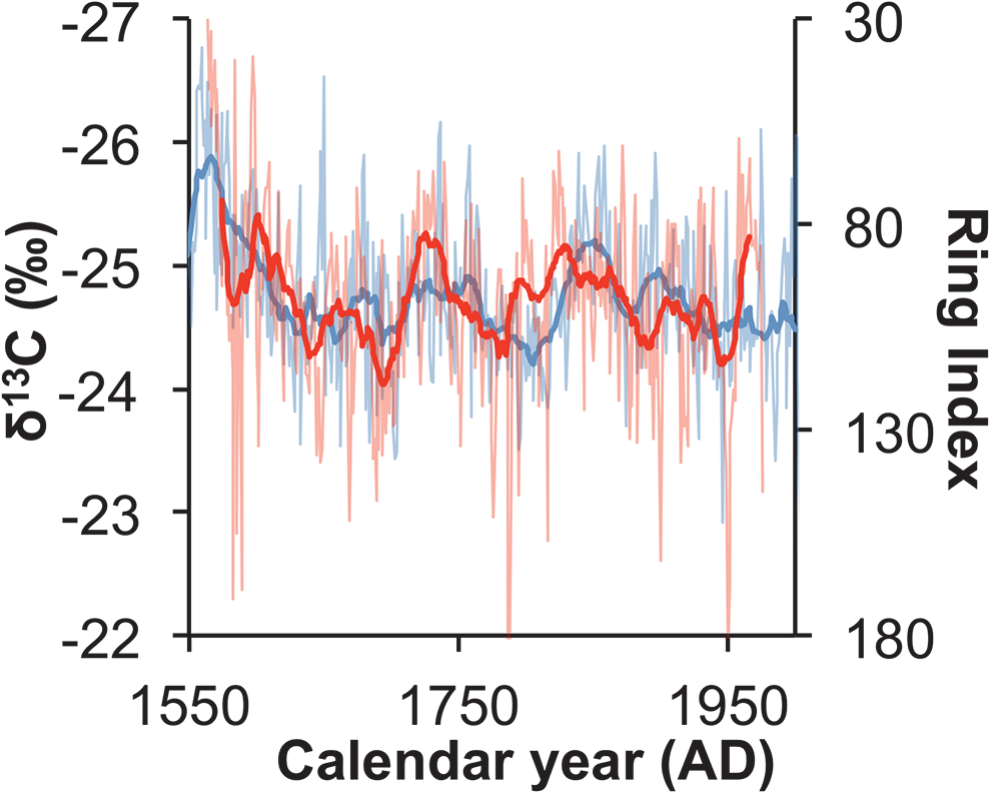
Summer and winter rainfall area proxy comparisons for southern Africa. The comparison between the δ^13^C baobab record (blue, left axis) and the cedar ring width record [32] (red, inverted right axis) demonstrates the existence of a rainfall dipole between the summer and winter rainfall areas of southern Africa.

Whereas the influence of ENSO and the DMI on southern African rainfall manifests in inter-annual to decadal zonal reorganization of the synoptic systems [4], they do not account for the latitudinal changes in climate systems in response to oceanic temperatures. Cooling of the Agulhas Current, as is demonstrated for the Little Ice Age in the Ifaty coral record, should lead to northward displacement of the westerlies [21, 22]. It is interesting to compare the baobab record with the cedar chronology from the winter rainfall area [32]. This comparison yields a significant correlation (r = 0.368, p<0.001, n = 374) (Fig 6) that suggests that relatively wet periods in the summer rainfall area correspond with relatively dry periods in the winter rainfall area. Precipitation in the winter rainfall area increases with a northward displacement of the westerlies. The inverse coupling between the cedar and baobab records suggests that this is accompanied by dryer conditions in the summer rainfall zone. The observed wet conditions in the winter rainfall area during the Little Ice Age [23, 25] would imply dry conditions in the summer rainfall area whereas a southward displacement of the austral summer location of the ITCZ should result in increased rainfall. Accordingly the high rainfall during the Medieval Warm Period transitioning to the low rainfall during the Little Ice Age in the baobab record is consistent with a northward displacement of the westerlies,

Most of the evidence for a northward shift of the westerlies in southern Africa during the Little Ice Age derives from the winter rainfall area, and it is only the Cold Air Cave speleothem record [24, 66] that currently provides evidence from the summer rainfall area. A negative δ^18^O excursion during the Little Ice Age is interpreted as increased high-level stratiform cloud formation and is associated with dryer conditions [24]. This interpretation rejects the alternative explanation that the pattern might be the result of a rainfall amount effect, which would suggest wetter conditions. The baobab evidence for dry conditions during the Little Ice Age is independent verification that the negative Cold Air cave speleothem δ^18^O excursion cannot be a rainfall amount effect. A high-resolution re-analysis of the T7 speleothem from Cold Air Cave [66] provides evidence that the δ^18^O record is a proxy for temperature, and that a c. 1.4°C cooling occurred during the Little Ice Age. The combined evidence is that the summer rainfall area of southern Africa was cooler and dryer during the Little Ice Age.

## Conclusions

A 1000-year, near-annually resolved proxy rainfall record from South African baobab trees shows decadal and centennial oscillations in rainfall with an overall decline in rainfall from AD 1075 till AD1805. Forcing of rainfall variability is a complex interaction between factors: The baobab record and the mukwa record from Zimbabwe indicate that absolute SST variability in the southwestern Indian Ocean is negatively correlated with rainfall in the region. The Indian Ocean DMI is also negatively correlated with rainfall. The widely accepted correlation between El Niño (la Nina) conditions in the tropical Pacific Ocean and dry (wet) conditions in the summer rainfall region of southern Africa is transient. Over the last 700 years El Niño (la Nina) conditions are generally correlated with wet (dry) conditions in the region. A relatively abrupt transition to dryer conditions takes place at c. AD 1600 and this is considered to be a regime shift. The rainfall dipole that emerges between the winter and summer rainfall areas suggests that the regime shift was caused by a northward displacement of the subtropical westerlies and not a southward displacement of the ITCZ.

## Supporting Information

S1 Fig Age models for baobab trees. (A) The ages assigned to isotope samples from core samples taken from baobab trees were determined from linear interpolations of core length (x-axis) with AMS radiocarbon dates. The 1-sigma radiocarbon error ranges are portrayed as vertical lines or as crosses (for bomb-carbon dates). (B) For trees that yielded ring structures the ring count (x-axis) matched the 1-simga AMS radiocarbon ages (vertical lines or crosses) with a 1:1 except where a buttress forms in one of the trees.
(TIFF)

S2 Fig The baobab isotope record and rainfall. Instrumental rainfall from the Pafuri station (orange, left axis) and the CRU3.20 rainfall for the region (red, left axis) cannot be correlated with the baobab δ^13^C record (blue, right axis) because of systemic errors in the age model.
(TIFF)

S3 Fig Comparison of ENSO proxy datasets. The ENSO proxy datasets of Li et al. [50] (dark blue), Cook et al. [51] (red), Stahle and Cleavland [52] (pale bue) and Braganza et al. [53] (purple) are coherent with the Niño3.4 index (http://www.cpc.ncep.noaa.gov/data/indices/sstoi.indices) (black). Only a 150-year section of the record is portrayed for clarity. Note that the indices of Briganza et al. and Stahle et al. are plotted on the inverted right axis because of the manner in which they are formulated.
(TIFF)

S4 Fig Oceanic sea-surface temperature trends. SST reconstructions for the Makassar Stait of Oppo et al. [64] (black, left axis) and Newton et al. [65] (blue, left axis) match the timing and relative temperature changes that took place in the Agulhas Current core region [55, 56] (red, right axis). The Ifaty record is shown along with the biweight mean value.
(TIFF)

S1 Table Correlation of baobab δ^13^C with environmental parameters. (DOCX)
