# Supplementary figures and images for: A 1000-Year Carbon Isotope Rainfall Proxy Record from South African Baobab Trees (Adansonia digitata L.)
Source: PLoS One. 2015 May 13;10(5):e0124202. doi: 10.1371/journal.pone.0124202 (PMC4430471; doi:10.1371/journal.pone.0124202)

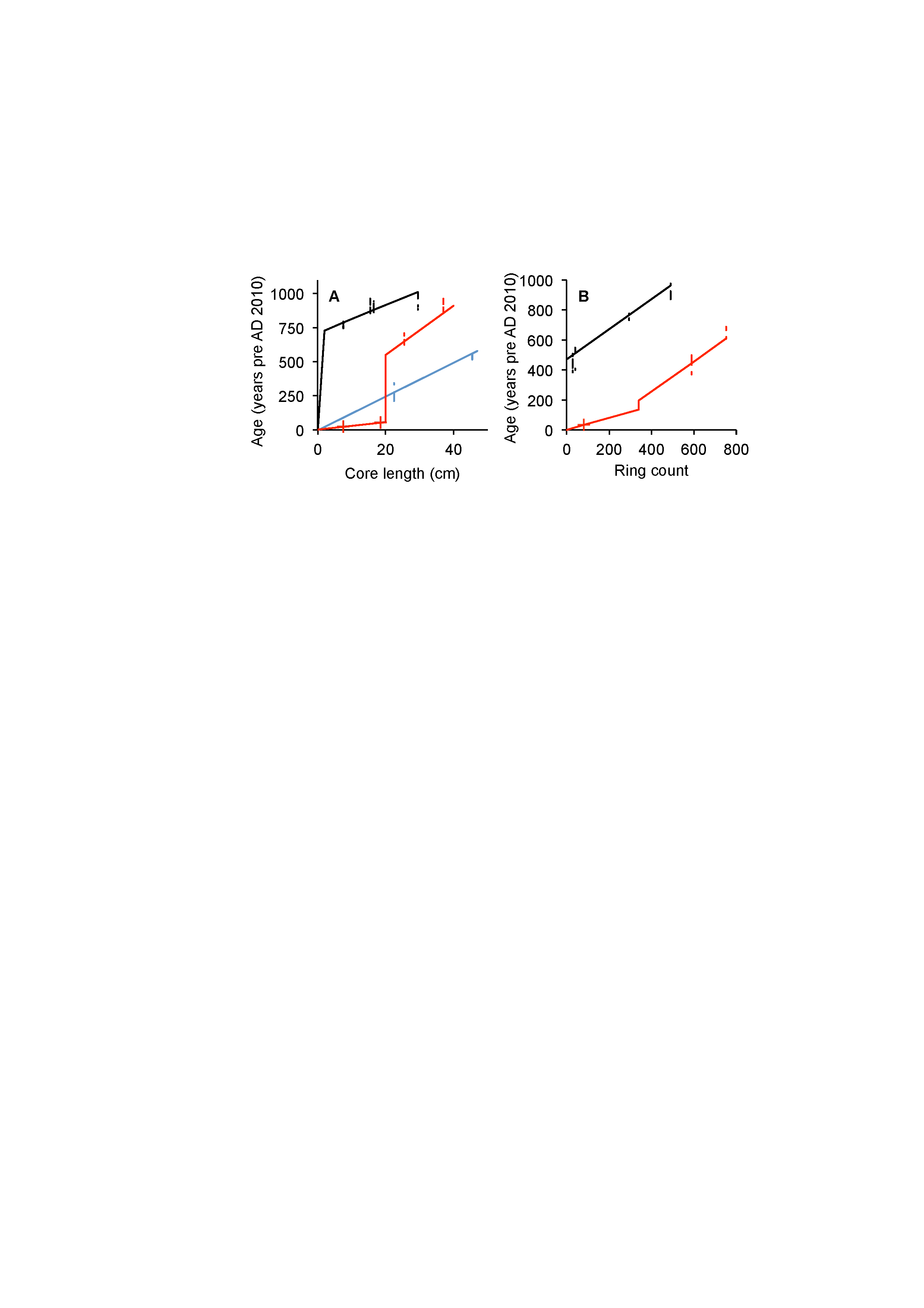

Supplement: S1 Fig — (A) The ages assigned to isotope samples from core samples taken from baobab trees were determined from linear interpolations of core length (x-axis) with AMS radiocarbon dates. The 1-sigma radiocarbon error ranges are portrayed as vertical lines or as crosses (for bomb-carbon dates). (B) For trees that yielded ring structures the ring count (x-axis) matched the 1-simga AMS radiocarbon ages (vertical lines or crosses) with a 1:1 except where a buttress forms in one of the trees. (TIFF) [file pone.0124202.s001.tiff]

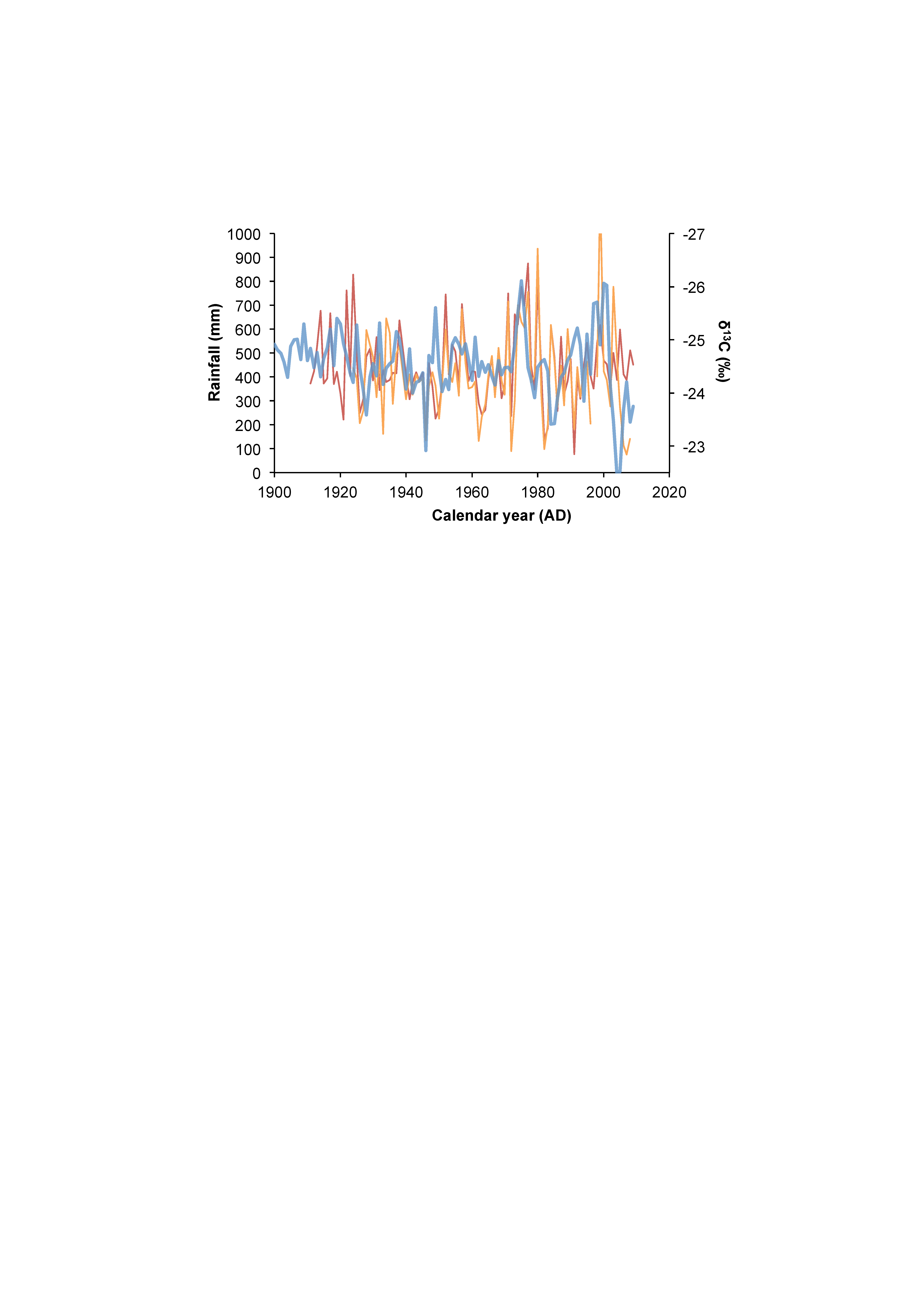

Supplement: S2 Fig — Instrumental rainfall from the Pafuri station (orange, left axis) and the CRU3.20 rainfall for the region (red, left axis) cannot be correlated with the baobab δ13C record (blue, right axis) because of systemic errors in the age model. (TIFF) [file pone.0124202.s002.tiff]

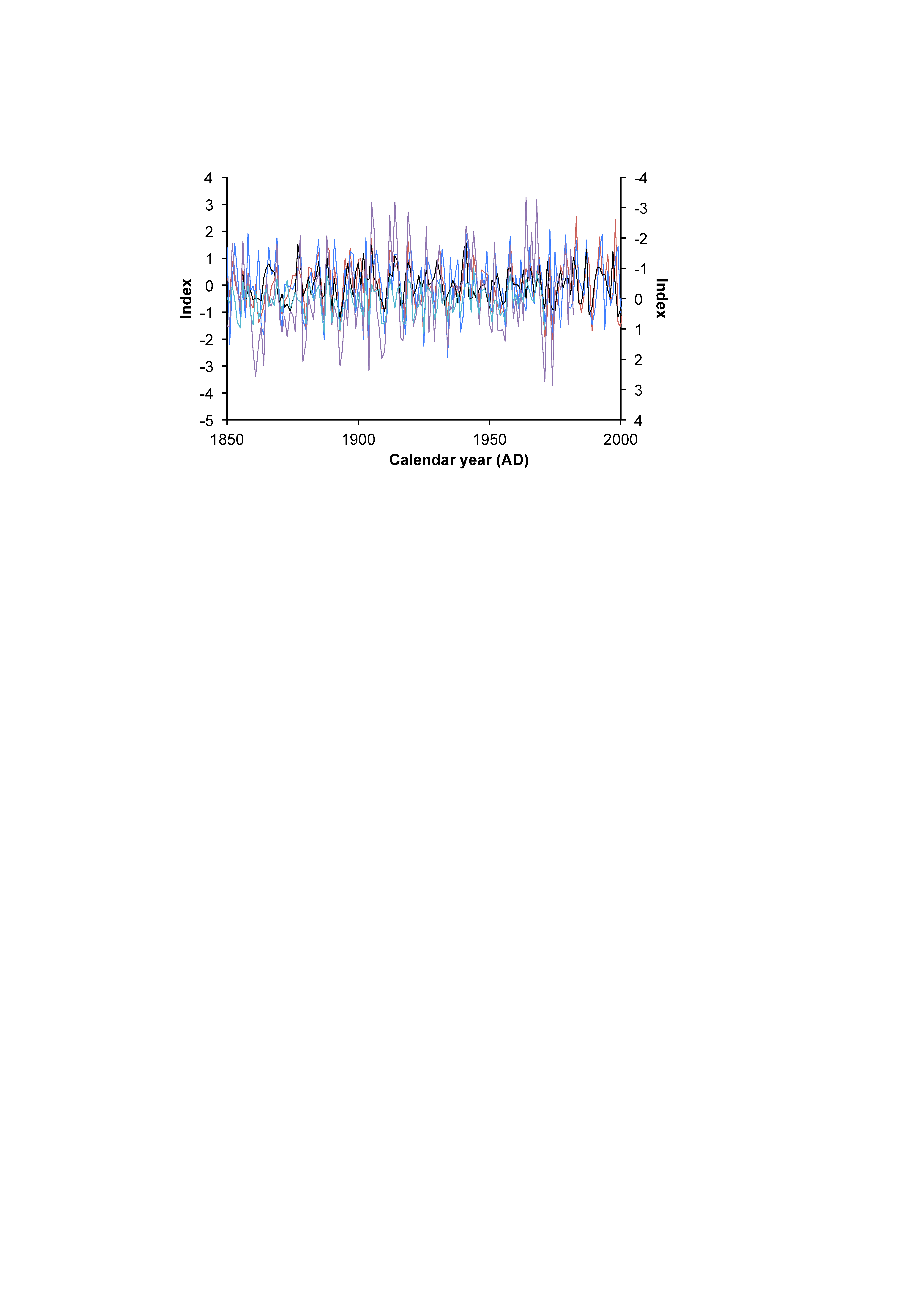

Supplement: S3 Fig — The ENSO proxy datasets of Li et al. [50] (dark blue), Cook et al. [51] (red), Stahle and Cleavland [52] (pale bue) and Braganza et al. [53] (purple) are coherent with the Niño3.4 index (http://www.cpc.ncep.noaa.gov/data/indices/sstoi.indices) (black). Only a 150-year section of the record is portrayed for clarity. Note that the indices of Briganza et al. and Stahle et al. are plotted on the inverted right axis because of the manner in which they are formulated. (TIFF) [file pone.0124202.s003.tiff]

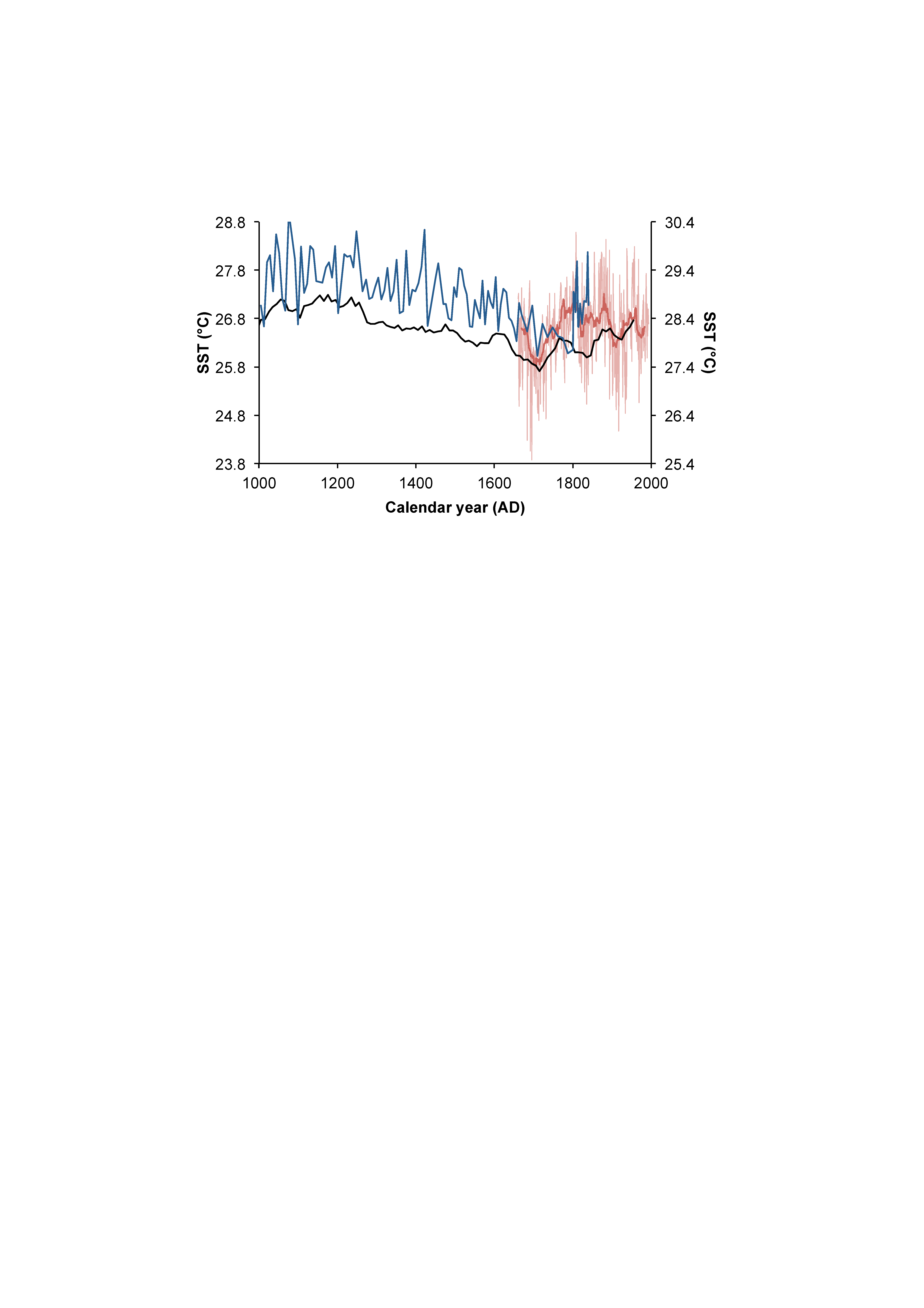

Supplement: S4 Fig — SST reconstructions for the Makassar Stait of Oppo et al. [64] (black, left axis) and Newton et al. [65] (blue, left axis) match the timing and relative temperature changes that took place in the Agulhas Current core region [55, 56] (red, right axis). The Ifaty record is shown along with the biweight mean value. (TIFF) [file pone.0124202.s004.tiff]
