# Supplementary material for: A 1000-Year Carbon Isotope Rainfall Proxy Record from South African Baobab Trees (Adansonia digitata L.)
Source: PLoS One. 2015 May 13;10(5):e0124202. doi: 10.1371/journal.pone.0124202 (PMC4430471; doi:10.1371/journal.pone.0124202)
Supplement: S1 Table — (DOCX) [file pone.0124202.s005.docx]

| **Parameter** | **Data source** | **Period** | **r** | **p** | **n** |
| --- | --- | --- | --- | --- | --- |
| Rainfall | Pafuri Instrumental | 1924-2008 | -0.246 | 0.024 | 84 |
|  | Neukom et al. [4] | 1806-1986 | -0.514 | <0.001 | 162 |
|  | Therrell et al. [29] | 1806-1986 | -0.112 | 0.154 | 162 |
|  |  | Post AD 1900 | -0.493 | <0.001 | 87 |
|  | Dunwidde & LaMarche [31] | 1574-1966 | 0.368 | <0.001 | 374 |
| SST | Zinke et al. [55] | 1670-1983 | 0.283 | <0.001 | 295 |
| DMI | Abram et al. [8] | 1858-1983 | 0.784 | <0.001 | 124 |
| ENSO | Li et al. [53] | 1300 - 1550 | -0.495 | <0.001 | 240 |
|  |  | 1710 -1970 | -0.428 | <0.001 | 242 |

Correlations are calculated using the KNMI Climate Explorer web site (<http://climexp.knmi.nl>).
